# Supplementary material for: Modeling glioblastoma heterogeneity as a dynamic network of cell states
Source: Mol Syst Biol. 2021 Sep 16;17(9):e10105. doi: 10.15252/msb.202010105 (PMC8444284; doi:10.15252/msb.202010105)
Supplement: Supplementary file 6 — Source Data for Figure 5 [file MSB-17-e10105-s004.zip › Figure5A_sourcedata/GSEA_3017/nosigdb_stateE.GseaPreranked.1622795031093/RICHARDS_NOWAKOWSKI_SCIENCE_2017_IPC_DIV2_UPREG.html]

Details for gene set RICHARDS\_NOWAKOWSKI\_SCIENCE\_2017\_IPC\_DIV2\_UPREG[GSEA]

|  || Dataset | state63017 |
| Phenotype | NoPhenotypeAvailable |
| Upregulated in class | na\_neg |
| GeneSet | RICHARDS\_NOWAKOWSKI\_SCIENCE\_2017\_IPC\_DIV2\_UPREG |
| Enrichment Score (ES) | -0.62342745 |
| Normalized Enrichment Score (NES) | -3.776295 |
| Nominal p-value | 0.0 |
| FDR q-value | 0.0 |
| FWER p-Value | 0.0 |
Table: GSEA Results Summary

  

Fig 1: Enrichment plot: RICHARDS\_NOWAKOWSKI\_SCIENCE\_2017\_IPC\_DIV2\_UPREG      
 Profile of the Running ES Score & Positions of GeneSet Members on the Rank Ordered List

  

| PROBE | GENE SYMBOL | GENE\_TITLE | RANK IN GENE LIST | RANK METRIC SCORE | RUNNING ES | CORE ENRICHMENT || 1 | TMSB4X |  |  | 93 | 0.412 | -0.1640 | No |
| 2 | SHROOM3 |  |  | 95 | 0.410 | -0.1638 | No |
| 3 | EZR |  |  | 96 | 0.410 | -0.1617 | No |
| 4 | STK17A |  |  | 97 | 0.408 | -0.1597 | No |
| 5 | EML1 |  |  | 110 | 0.383 | -0.1792 | No |
| 6 | FAM200B |  |  | 120 | 0.361 | -0.1935 | No |
| 7 | DAAM1 |  |  | 138 | 0.343 | -0.2222 | No |
| 8 | LZTS1 |  |  | 187 | 0.306 | -0.3064 | No |
| 9 | CUX1 |  |  | 188 | 0.306 | -0.3049 | No |
| 10 | TMEM47 |  |  | 222 | 0.282 | -0.3624 | No |
| 11 | SDC2 |  |  | 226 | 0.280 | -0.3664 | No |
| 12 | DAG1 |  |  | 228 | 0.279 | -0.3668 | No |
| 13 | GUK1 |  |  | 231 | 0.277 | -0.3690 | No |
| 14 | CDK6 |  |  | 238 | 0.269 | -0.3783 | No |
| 15 | CSRP2 |  |  | 247 | 0.264 | -0.3913 | No |
| 16 | TNPO2 |  |  | 273 | -0.251 | -0.4347 | No |
| 17 | SUPT16H |  |  | 276 | -0.251 | -0.4370 | No |
| 18 | ARHGAP11B |  |  | 277 | -0.252 | -0.4358 | No |
| 19 | FIGNL1 |  |  | 290 | -0.257 | -0.4559 | No |
| 20 | ZNF850 |  |  | 291 | -0.257 | -0.4547 | No |
| 21 | RTTN |  |  | 295 | -0.257 | -0.4588 | No |
| 22 | DOT1L |  |  | 296 | -0.257 | -0.4575 | No |
| 23 | H2AFY |  |  | 297 | -0.257 | -0.4562 | No |
| 24 | RAN |  |  | 298 | -0.257 | -0.4549 | No |
| 25 | DAZAP1 |  |  | 299 | -0.258 | -0.4536 | No |
| 26 | YEATS4 |  |  | 300 | -0.258 | -0.4524 | No |
| 27 | RNF168 |  |  | 301 | -0.258 | -0.4511 | No |
| 28 | ERI1 |  |  | 302 | -0.258 | -0.4498 | No |
| 29 | WDR34 |  |  | 303 | -0.258 | -0.4485 | No |
| 30 | PRKD3 |  |  | 304 | -0.258 | -0.4472 | No |
| 31 | ZNF682 |  |  | 309 | -0.260 | -0.4531 | No |
| 32 | AHI1 |  |  | 310 | -0.261 | -0.4518 | No |
| 33 | HNRNPAB |  |  | 312 | -0.261 | -0.4523 | No |
| 34 | TGIF1 |  |  | 318 | -0.263 | -0.4599 | No |
| 35 | TUBG1 |  |  | 323 | -0.263 | -0.4658 | No |
| 36 | CA12 |  |  | 325 | -0.263 | -0.4662 | No |
| 37 | CNTLN |  |  | 326 | -0.264 | -0.4649 | No |
| 38 | MAGOHB |  |  | 328 | -0.264 | -0.4654 | No |
| 39 | PAICS |  |  | 336 | -0.265 | -0.4766 | No |
| 40 | MCM5 |  |  | 337 | -0.265 | -0.4753 | No |
| 41 | KNTC1 |  |  | 352 | -0.269 | -0.4989 | No |
| 42 | NUDT15 |  |  | 356 | -0.270 | -0.5030 | No |
| 43 | STAG1 |  |  | 360 | -0.271 | -0.5070 | No |
| 44 | ARHGAP33 |  |  | 363 | -0.272 | -0.5092 | No |
| 45 | DLEU2 |  |  | 365 | -0.272 | -0.5096 | No |
| 46 | TBC1D31 |  |  | 380 | -0.276 | -0.5333 | No |
| 47 | IVNS1ABP |  |  | 381 | -0.276 | -0.5319 | No |
| 48 | RNF165 |  |  | 382 | -0.276 | -0.5305 | No |
| 49 | TMEM237 |  |  | 383 | -0.276 | -0.5291 | No |
| 50 | SLBP |  |  | 386 | -0.277 | -0.5313 | No |
| 51 | HNRNPA2B1 |  |  | 391 | -0.279 | -0.5371 | No |
| 52 | CTBP2 |  |  | 393 | -0.280 | -0.5375 | No |
| 53 | NFATC2IP |  |  | 397 | -0.283 | -0.5415 | No |
| 54 | SNRPG |  |  | 398 | -0.283 | -0.5401 | No |
| 55 | PPP2R3C |  |  | 400 | -0.283 | -0.5404 | No |
| 56 | DERA |  |  | 402 | -0.284 | -0.5408 | No |
| 57 | LSM4 |  |  | 406 | -0.284 | -0.5448 | No |
| 58 | NASP |  |  | 410 | -0.285 | -0.5487 | No |
| 59 | SIVA1 |  |  | 412 | -0.286 | -0.5491 | No |
| 60 | MAGI1 |  |  | 413 | -0.287 | -0.5476 | No |
| 61 | CDK2 |  |  | 422 | -0.290 | -0.5605 | No |
| 62 | CHCHD2 |  |  | 423 | -0.290 | -0.5591 | No |
| 63 | FANCG |  |  | 424 | -0.291 | -0.5576 | No |
| 64 | EGFR |  |  | 427 | -0.291 | -0.5597 | No |
| 65 | SEPT10 |  |  | 431 | -0.293 | -0.5636 | No |
| 66 | ZNF738 |  |  | 437 | -0.295 | -0.5711 | No |
| 67 | HMGXB4 |  |  | 438 | -0.296 | -0.5696 | No |
| 68 | LSM8 |  |  | 441 | -0.297 | -0.5717 | No |
| 69 | PPIF |  |  | 442 | -0.297 | -0.5703 | No |
| 70 | CTCF |  |  | 443 | -0.297 | -0.5688 | No |
| 71 | KPNB1 |  |  | 445 | -0.298 | -0.5691 | No |
| 72 | PLCB1 |  |  | 446 | -0.298 | -0.5676 | No |
| 73 | ZNF43 |  |  | 449 | -0.299 | -0.5697 | No |
| 74 | CDC7 |  |  | 451 | -0.299 | -0.5700 | No |
| 75 | HNRNPD |  |  | 456 | -0.301 | -0.5757 | No |
| 76 | MCM4 |  |  | 457 | -0.301 | -0.5742 | No |
| 77 | UBR7 |  |  | 459 | -0.302 | -0.5745 | No |
| 78 | CNTRL |  |  | 462 | -0.304 | -0.5765 | No |
| 79 | DNMT1 |  |  | 466 | -0.304 | -0.5804 | No |
| 80 | SUV39H2 |  |  | 469 | -0.305 | -0.5824 | No |
| 81 | SMC1A |  |  | 472 | -0.307 | -0.5845 | No |
| 82 | NUMA1 |  |  | 477 | -0.307 | -0.5901 | No |
| 83 | EXOSC9 |  |  | 481 | -0.308 | -0.5939 | No |
| 84 | NOP56 |  |  | 483 | -0.309 | -0.5942 | No |
| 85 | HIP1 |  |  | 484 | -0.309 | -0.5926 | No |
| 86 | PRDX3 |  |  | 489 | -0.310 | -0.5982 | No |
| 87 | NEDD1 |  |  | 491 | -0.311 | -0.5985 | No |
| 88 | RSRC1 |  |  | 494 | -0.312 | -0.6005 | No |
| 89 | NUP205 |  |  | 495 | -0.313 | -0.5990 | No |
| 90 | ILF2 |  |  | 497 | -0.313 | -0.5992 | No |
| 91 | RFC2 |  |  | 498 | -0.313 | -0.5976 | No |
| 92 | CEP97 |  |  | 499 | -0.313 | -0.5961 | No |
| 93 | PA2G4 |  |  | 501 | -0.314 | -0.5963 | No |
| 94 | GGH |  |  | 504 | -0.314 | -0.5983 | No |
| 95 | COX17 |  |  | 505 | -0.316 | -0.5968 | No |
| 96 | HNRNPH3 |  |  | 507 | -0.316 | -0.5970 | No |
| 97 | PIK3R3 |  |  | 508 | -0.317 | -0.5954 | No |
| 98 | SRRT |  |  | 509 | -0.317 | -0.5938 | No |
| 99 | BARD1 |  |  | 510 | -0.318 | -0.5923 | No |
| 100 | NSMCE4A |  |  | 511 | -0.318 | -0.5907 | No |
| 101 | DNA2 |  |  | 513 | -0.318 | -0.5909 | No |
| 102 | CCDC150 |  |  | 516 | -0.319 | -0.5929 | No |
| 103 | HAUS1 |  |  | 518 | -0.320 | -0.5931 | No |
| 104 | KIF20A |  |  | 523 | -0.321 | -0.5986 | No |
| 105 | SUZ12 |  |  | 524 | -0.322 | -0.5970 | No |
| 106 | RHEB |  |  | 528 | -0.323 | -0.6008 | No |
| 107 | NUP62 |  |  | 529 | -0.323 | -0.5992 | No |
| 108 | LSM3 |  |  | 530 | -0.323 | -0.5976 | No |
| 109 | CEP192 |  |  | 531 | -0.324 | -0.5960 | No |
| 110 | CEP135 |  |  | 532 | -0.325 | -0.5944 | No |
| 111 | ACTL6A |  |  | 533 | -0.325 | -0.5928 | No |
| 112 | RFC5 |  |  | 534 | -0.326 | -0.5911 | No |
| 113 | SNHG1 |  |  | 538 | -0.328 | -0.5949 | No |
| 114 | PRIM1 |  |  | 539 | -0.328 | -0.5932 | No |
| 115 | H3F3A |  |  | 542 | -0.329 | -0.5952 | No |
| 116 | RBM17 |  |  | 546 | -0.331 | -0.5989 | No |
| 117 | GLRX3 |  |  | 547 | -0.332 | -0.5973 | No |
| 118 | MSH2 |  |  | 548 | -0.332 | -0.5956 | No |
| 119 | CDC25C |  |  | 549 | -0.332 | -0.5940 | No |
| 120 | PLK4 |  |  | 551 | -0.334 | -0.5941 | No |
| 121 | EIF4EBP2 |  |  | 552 | -0.335 | -0.5924 | No |
| 122 | ZNF90 |  |  | 557 | -0.338 | -0.5979 | No |
| 123 | MCM3 |  |  | 560 | -0.340 | -0.5998 | No |
| 124 | HAT1 |  |  | 561 | -0.340 | -0.5981 | No |
| 125 | CEP128 |  |  | 565 | -0.342 | -0.6018 | No |
| 126 | LSM5 |  |  | 568 | -0.342 | -0.6036 | No |
| 127 | HMGB3 |  |  | 577 | -0.348 | -0.6162 | No |
| 128 | RCC1 |  |  | 582 | -0.350 | -0.6216 | No |
| 129 | GEN1 |  |  | 584 | -0.351 | -0.6216 | No |
| 130 | NUP107 |  |  | 586 | -0.352 | -0.6217 | Yes |
| 131 | DSCC1 |  |  | 587 | -0.352 | -0.6199 | Yes |
| 132 | DLL1 |  |  | 588 | -0.352 | -0.6182 | Yes |
| 133 | DDX39A |  |  | 590 | -0.353 | -0.6182 | Yes |
| 134 | CDK5RAP2 |  |  | 591 | -0.353 | -0.6165 | Yes |
| 135 | EIF4EBP1 |  |  | 592 | -0.353 | -0.6147 | Yes |
| 136 | CMSS1 |  |  | 593 | -0.353 | -0.6130 | Yes |
| 137 | CENPC |  |  | 596 | -0.356 | -0.6148 | Yes |
| 138 | GGCT |  |  | 598 | -0.356 | -0.6148 | Yes |
| 139 | RNASEH2B |  |  | 599 | -0.356 | -0.6130 | Yes |
| 140 | COMMD4 |  |  | 601 | -0.358 | -0.6130 | Yes |
| 141 | HIRIP3 |  |  | 603 | -0.359 | -0.6131 | Yes |
| 142 | FANCB |  |  | 606 | -0.361 | -0.6148 | Yes |
| 143 | PRIM2 |  |  | 607 | -0.362 | -0.6130 | Yes |
| 144 | ASRGL1 |  |  | 608 | -0.362 | -0.6112 | Yes |
| 145 | PARP2 |  |  | 609 | -0.362 | -0.6094 | Yes |
| 146 | WDHD1 |  |  | 610 | -0.363 | -0.6076 | Yes |
| 147 | KIF18B |  |  | 612 | -0.363 | -0.6076 | Yes |
| 148 | PRTFDC1 |  |  | 613 | -0.364 | -0.6058 | Yes |
| 149 | BTG3 |  |  | 615 | -0.365 | -0.6058 | Yes |
| 150 | RFWD3 |  |  | 617 | -0.365 | -0.6058 | Yes |
| 151 | RPP30 |  |  | 618 | -0.365 | -0.6040 | Yes |
| 152 | C18orf54 |  |  | 619 | -0.366 | -0.6022 | Yes |
| 153 | ZGRF1 |  |  | 620 | -0.367 | -0.6003 | Yes |
| 154 | HNRNPF |  |  | 621 | -0.369 | -0.5985 | Yes |
| 155 | DDX11 |  |  | 622 | -0.370 | -0.5967 | Yes |
| 156 | WDR76 |  |  | 626 | -0.373 | -0.6002 | Yes |
| 157 | NEK2 |  |  | 628 | -0.374 | -0.6001 | Yes |
| 158 | EXOSC8 |  |  | 630 | -0.376 | -0.6000 | Yes |
| 159 | SAMD1 |  |  | 632 | -0.377 | -0.5999 | Yes |
| 160 | RNASEH2A |  |  | 633 | -0.378 | -0.5981 | Yes |
| 161 | RPS24 |  |  | 635 | -0.381 | -0.5980 | Yes |
| 162 | CCP110 |  |  | 636 | -0.381 | -0.5961 | Yes |
| 163 | RMI1 |  |  | 637 | -0.381 | -0.5942 | Yes |
| 164 | SEZ6L |  |  | 642 | -0.383 | -0.5994 | Yes |
| 165 | PHIP |  |  | 643 | -0.384 | -0.5975 | Yes |
| 166 | MPHOSPH9 |  |  | 644 | -0.384 | -0.5956 | Yes |
| 167 | TTF2 |  |  | 646 | -0.386 | -0.5955 | Yes |
| 168 | PHF19 |  |  | 648 | -0.391 | -0.5953 | Yes |
| 169 | POLE |  |  | 652 | -0.395 | -0.5987 | Yes |
| 170 | ZEB1 |  |  | 653 | -0.396 | -0.5968 | Yes |
| 171 | TMEM106C |  |  | 654 | -0.397 | -0.5948 | Yes |
| 172 | SHCBP1 |  |  | 655 | -0.398 | -0.5928 | Yes |
| 173 | GINS2 |  |  | 658 | -0.399 | -0.5944 | Yes |
| 174 | PBX3 |  |  | 662 | -0.403 | -0.5978 | Yes |
| 175 | RHNO1 |  |  | 664 | -0.406 | -0.5976 | Yes |
| 176 | MNS1 |  |  | 666 | -0.408 | -0.5973 | Yes |
| 177 | SAE1 |  |  | 668 | -0.408 | -0.5971 | Yes |
| 178 | PGAM1 |  |  | 670 | -0.410 | -0.5968 | Yes |
| 179 | DSN1 |  |  | 671 | -0.410 | -0.5948 | Yes |
| 180 | SOGA1 |  |  | 672 | -0.411 | -0.5928 | Yes |
| 181 | KIF18A |  |  | 673 | -0.412 | -0.5907 | Yes |
| 182 | NUDT1 |  |  | 674 | -0.413 | -0.5887 | Yes |
| 183 | GNG4 |  |  | 676 | -0.415 | -0.5884 | Yes |
| 184 | NUP50 |  |  | 677 | -0.415 | -0.5863 | Yes |
| 185 | CIT |  |  | 680 | -0.418 | -0.5878 | Yes |
| 186 | RBMX |  |  | 682 | -0.421 | -0.5875 | Yes |
| 187 | H2AFV |  |  | 684 | -0.423 | -0.5872 | Yes |
| 188 | CCDC14 |  |  | 685 | -0.423 | -0.5851 | Yes |
| 189 | CENPO |  |  | 686 | -0.424 | -0.5830 | Yes |
| 190 | CDCA4 |  |  | 687 | -0.424 | -0.5809 | Yes |
| 191 | RPA2 |  |  | 688 | -0.424 | -0.5788 | Yes |
| 192 | CENPQ |  |  | 689 | -0.425 | -0.5767 | Yes |
| 193 | FANCA |  |  | 691 | -0.425 | -0.5764 | Yes |
| 194 | STIL |  |  | 692 | -0.425 | -0.5743 | Yes |
| 195 | SLC25A5 |  |  | 695 | -0.426 | -0.5757 | Yes |
| 196 | DIAPH3 |  |  | 696 | -0.427 | -0.5736 | Yes |
| 197 | RANBP1 |  |  | 697 | -0.427 | -0.5715 | Yes |
| 198 | ANP32B |  |  | 699 | -0.431 | -0.5712 | Yes |
| 199 | E2F3 |  |  | 700 | -0.431 | -0.5690 | Yes |
| 200 | CHEK1 |  |  | 704 | -0.433 | -0.5722 | Yes |
| 201 | PARPBP |  |  | 705 | -0.434 | -0.5701 | Yes |
| 202 | TIMELESS |  |  | 707 | -0.435 | -0.5697 | Yes |
| 203 | TEX30 |  |  | 709 | -0.436 | -0.5693 | Yes |
| 204 | POLD3 |  |  | 710 | -0.437 | -0.5672 | Yes |
| 205 | MZT1 |  |  | 711 | -0.437 | -0.5650 | Yes |
| 206 | ZNF93 |  |  | 712 | -0.438 | -0.5628 | Yes |
| 207 | LMNB2 |  |  | 713 | -0.438 | -0.5606 | Yes |
| 208 | RBBP8 |  |  | 714 | -0.440 | -0.5585 | Yes |
| 209 | RAD18 |  |  | 715 | -0.443 | -0.5563 | Yes |
| 210 | ALYREF |  |  | 716 | -0.444 | -0.5541 | Yes |
| 211 | SLC20A1 |  |  | 717 | -0.444 | -0.5519 | Yes |
| 212 | SKA1 |  |  | 719 | -0.447 | -0.5514 | Yes |
| 213 | PSAT1 |  |  | 720 | -0.448 | -0.5492 | Yes |
| 214 | MASTL |  |  | 721 | -0.451 | -0.5470 | Yes |
| 215 | MMP16 |  |  | 723 | -0.454 | -0.5465 | Yes |
| 216 | TTK |  |  | 724 | -0.454 | -0.5443 | Yes |
| 217 | PCNT |  |  | 725 | -0.454 | -0.5420 | Yes |
| 218 | POLQ |  |  | 726 | -0.455 | -0.5397 | Yes |
| 219 | LINC01224 |  |  | 727 | -0.455 | -0.5375 | Yes |
| 220 | RAD21 |  |  | 728 | -0.458 | -0.5352 | Yes |
| 221 | SPAG5 |  |  | 729 | -0.458 | -0.5329 | Yes |
| 222 | GINS1 |  |  | 730 | -0.459 | -0.5307 | Yes |
| 223 | CCNF |  |  | 732 | -0.460 | -0.5302 | Yes |
| 224 | POLA1 |  |  | 733 | -0.460 | -0.5279 | Yes |
| 225 | BRIP1 |  |  | 734 | -0.461 | -0.5256 | Yes |
| 226 | ZNF714 |  |  | 735 | -0.461 | -0.5233 | Yes |
| 227 | CSE1L |  |  | 736 | -0.461 | -0.5210 | Yes |
| 228 | C19orf48 |  |  | 738 | -0.462 | -0.5205 | Yes |
| 229 | LBR |  |  | 739 | -0.463 | -0.5182 | Yes |
| 230 | E2F8 |  |  | 741 | -0.467 | -0.5177 | Yes |
| 231 | MGME1 |  |  | 742 | -0.468 | -0.5154 | Yes |
| 232 | SMC3 |  |  | 743 | -0.470 | -0.5130 | Yes |
| 233 | NUCKS1 |  |  | 744 | -0.471 | -0.5107 | Yes |
| 234 | PTMA |  |  | 745 | -0.475 | -0.5083 | Yes |
| 235 | CHD7 |  |  | 746 | -0.480 | -0.5060 | Yes |
| 236 | TK1 |  |  | 747 | -0.480 | -0.5036 | Yes |
| 237 | MIS18A |  |  | 750 | -0.483 | -0.5048 | Yes |
| 238 | LIG1 |  |  | 752 | -0.485 | -0.5041 | Yes |
| 239 | PRR11 |  |  | 755 | -0.488 | -0.5053 | Yes |
| 240 | CDCA3 |  |  | 759 | -0.497 | -0.5082 | Yes |
| 241 | MMS22L |  |  | 761 | -0.500 | -0.5075 | Yes |
| 242 | DCP2 |  |  | 762 | -0.500 | -0.5050 | Yes |
| 243 | GMNN |  |  | 766 | -0.510 | -0.5078 | Yes |
| 244 | RTKN2 |  |  | 767 | -0.511 | -0.5053 | Yes |
| 245 | LRR1 |  |  | 768 | -0.512 | -0.5028 | Yes |
| 246 | CMC2 |  |  | 771 | -0.518 | -0.5038 | Yes |
| 247 | CDC45 |  |  | 773 | -0.521 | -0.5030 | Yes |
| 248 | DTYMK |  |  | 774 | -0.522 | -0.5004 | Yes |
| 249 | RFC4 |  |  | 777 | -0.527 | -0.5013 | Yes |
| 250 | DEPDC1B |  |  | 779 | -0.533 | -0.5005 | Yes |
| 251 | SKA3 |  |  | 783 | -0.539 | -0.5032 | Yes |
| 252 | NCAPD2 |  |  | 784 | -0.540 | -0.5005 | Yes |
| 253 | CEP152 |  |  | 785 | -0.542 | -0.4978 | Yes |
| 254 | BLM |  |  | 786 | -0.543 | -0.4951 | Yes |
| 255 | FANCD2 |  |  | 787 | -0.544 | -0.4924 | Yes |
| 256 | RPA3 |  |  | 788 | -0.550 | -0.4897 | Yes |
| 257 | INCENP |  |  | 789 | -0.558 | -0.4869 | Yes |
| 258 | NCAPD3 |  |  | 790 | -0.559 | -0.4841 | Yes |
| 259 | ATAD5 |  |  | 791 | -0.559 | -0.4814 | Yes |
| 260 | HAUS8 |  |  | 792 | -0.560 | -0.4786 | Yes |
| 261 | HMGN2 |  |  | 795 | -0.564 | -0.4794 | Yes |
| 262 | RACGAP1 |  |  | 796 | -0.566 | -0.4765 | Yes |
| 263 | HMGB1 |  |  | 797 | -0.567 | -0.4737 | Yes |
| 264 | ASF1B |  |  | 798 | -0.568 | -0.4709 | Yes |
| 265 | CENPJ |  |  | 799 | -0.568 | -0.4681 | Yes |
| 266 | HES6 |  |  | 800 | -0.569 | -0.4653 | Yes |
| 267 | RFC3 |  |  | 801 | -0.569 | -0.4625 | Yes |
| 268 | TUBB |  |  | 802 | -0.570 | -0.4596 | Yes |
| 269 | CEP78 |  |  | 803 | -0.570 | -0.4568 | Yes |
| 270 | CENPN |  |  | 805 | -0.574 | -0.4557 | Yes |
| 271 | USP1 |  |  | 806 | -0.575 | -0.4529 | Yes |
| 272 | CDC6 |  |  | 807 | -0.584 | -0.4500 | Yes |
| 273 | CDCA8 |  |  | 808 | -0.585 | -0.4471 | Yes |
| 274 | PLK1 |  |  | 809 | -0.586 | -0.4442 | Yes |
| 275 | CHAF1A |  |  | 810 | -0.588 | -0.4413 | Yes |
| 276 | PSMC3IP |  |  | 811 | -0.588 | -0.4383 | Yes |
| 277 | DEK |  |  | 812 | -0.591 | -0.4354 | Yes |
| 278 | FOXM1 |  |  | 814 | -0.593 | -0.4343 | Yes |
| 279 | CDCA2 |  |  | 816 | -0.597 | -0.4331 | Yes |
| 280 | MCM7 |  |  | 817 | -0.599 | -0.4301 | Yes |
| 281 | LMNB1 |  |  | 818 | -0.606 | -0.4271 | Yes |
| 282 | MCM8 |  |  | 819 | -0.607 | -0.4241 | Yes |
| 283 | MND1 |  |  | 821 | -0.615 | -0.4228 | Yes |
| 284 | CKAP2L |  |  | 826 | -0.625 | -0.4269 | Yes |
| 285 | CCDC34 |  |  | 827 | -0.626 | -0.4238 | Yes |
| 286 | DNAJC9 |  |  | 828 | -0.633 | -0.4206 | Yes |
| 287 | AURKB |  |  | 829 | -0.634 | -0.4175 | Yes |
| 288 | HELLS |  |  | 830 | -0.635 | -0.4143 | Yes |
| 289 | DTL |  |  | 831 | -0.637 | -0.4112 | Yes |
| 290 | PCNA |  |  | 832 | -0.640 | -0.4080 | Yes |
| 291 | BUB3 |  |  | 834 | -0.641 | -0.4066 | Yes |
| 292 | CENPW |  |  | 835 | -0.643 | -0.4034 | Yes |
| 293 | CDKN3 |  |  | 836 | -0.653 | -0.4002 | Yes |
| 294 | BUB1 |  |  | 837 | -0.653 | -0.3969 | Yes |
| 295 | ITGB3BP |  |  | 838 | -0.661 | -0.3937 | Yes |
| 296 | E2F7 |  |  | 840 | -0.675 | -0.3921 | Yes |
| 297 | ANP32E |  |  | 841 | -0.676 | -0.3887 | Yes |
| 298 | KIF22 |  |  | 842 | -0.678 | -0.3854 | Yes |
| 299 | BIRC5 |  |  | 843 | -0.679 | -0.3820 | Yes |
| 300 | CENPM |  |  | 844 | -0.682 | -0.3786 | Yes |
| 301 | NCAPH |  |  | 845 | -0.685 | -0.3752 | Yes |
| 302 | BRCA1 |  |  | 846 | -0.691 | -0.3718 | Yes |
| 303 | ZWINT |  |  | 847 | -0.692 | -0.3684 | Yes |
| 304 | SPC24 |  |  | 848 | -0.694 | -0.3649 | Yes |
| 305 | CENPH |  |  | 849 | -0.694 | -0.3615 | Yes |
| 306 | BRCA2 |  |  | 850 | -0.694 | -0.3580 | Yes |
| 307 | DHFR |  |  | 851 | -0.701 | -0.3546 | Yes |
| 308 | VRK1 |  |  | 852 | -0.711 | -0.3510 | Yes |
| 309 | ECT2 |  |  | 854 | -0.717 | -0.3493 | Yes |
| 310 | KIF2C |  |  | 855 | -0.721 | -0.3457 | Yes |
| 311 | NUF2 |  |  | 856 | -0.721 | -0.3421 | Yes |
| 312 | CENPK |  |  | 857 | -0.722 | -0.3385 | Yes |
| 313 | FAM111A |  |  | 858 | -0.727 | -0.3349 | Yes |
| 314 | SYNE2 |  |  | 859 | -0.728 | -0.3313 | Yes |
| 315 | ANLN |  |  | 860 | -0.733 | -0.3277 | Yes |
| 316 | KIF4A |  |  | 861 | -0.736 | -0.3240 | Yes |
| 317 | FEN1 |  |  | 862 | -0.736 | -0.3204 | Yes |
| 318 | FBXO5 |  |  | 864 | -0.742 | -0.3185 | Yes |
| 319 | CCNB2 |  |  | 865 | -0.745 | -0.3148 | Yes |
| 320 | RRM1 |  |  | 866 | -0.749 | -0.3111 | Yes |
| 321 | ARHGAP11A |  |  | 867 | -0.756 | -0.3073 | Yes |
| 322 | NDC80 |  |  | 869 | -0.762 | -0.3053 | Yes |
| 323 | ORC6 |  |  | 870 | -0.766 | -0.3015 | Yes |
| 324 | BUB1B |  |  | 871 | -0.766 | -0.2977 | Yes |
| 325 | CCNA2 |  |  | 872 | -0.770 | -0.2939 | Yes |
| 326 | KIF23 |  |  | 873 | -0.772 | -0.2901 | Yes |
| 327 | MELK |  |  | 874 | -0.776 | -0.2862 | Yes |
| 328 | TMPO |  |  | 875 | -0.778 | -0.2824 | Yes |
| 329 | KIF20B |  |  | 876 | -0.779 | -0.2785 | Yes |
| 330 | CKAP2 |  |  | 878 | -0.781 | -0.2764 | Yes |
| 331 | NCAPG2 |  |  | 879 | -0.781 | -0.2725 | Yes |
| 332 | PTTG1 |  |  | 880 | -0.791 | -0.2686 | Yes |
| 333 | MCM10 |  |  | 881 | -0.798 | -0.2647 | Yes |
| 334 | H2AFX |  |  | 882 | -0.799 | -0.2607 | Yes |
| 335 | KIFC1 |  |  | 883 | -0.807 | -0.2567 | Yes |
| 336 | KIF14 |  |  | 885 | -0.809 | -0.2545 | Yes |
| 337 | EZH2 |  |  | 886 | -0.810 | -0.2505 | Yes |
| 338 | FAM111B |  |  | 888 | -0.822 | -0.2482 | Yes |
| 339 | KIF15 |  |  | 890 | -0.834 | -0.2458 | Yes |
| 340 | CDCA5 |  |  | 892 | -0.841 | -0.2434 | Yes |
| 341 | H2AFZ |  |  | 893 | -0.849 | -0.2392 | Yes |
| 342 | RAD51AP1 |  |  | 894 | -0.853 | -0.2350 | Yes |
| 343 | KPNA2 |  |  | 895 | -0.858 | -0.2307 | Yes |
| 344 | DBF4 |  |  | 896 | -0.864 | -0.2264 | Yes |
| 345 | SMC2 |  |  | 897 | -0.867 | -0.2221 | Yes |
| 346 | CENPE |  |  | 899 | -0.873 | -0.2196 | Yes |
| 347 | PBK |  |  | 900 | -0.880 | -0.2152 | Yes |
| 348 | FANCI |  |  | 901 | -0.882 | -0.2109 | Yes |
| 349 | SMC4 |  |  | 902 | -0.886 | -0.2065 | Yes |
| 350 | DUT |  |  | 903 | -0.887 | -0.2021 | Yes |
| 351 | MIS18BP1 |  |  | 904 | -0.889 | -0.1976 | Yes |
| 352 | C21orf58 |  |  | 905 | -0.893 | -0.1932 | Yes |
| 353 | CCNE2 |  |  | 907 | -0.895 | -0.1906 | Yes |
| 354 | XRCC2 |  |  | 908 | -0.907 | -0.1861 | Yes |
| 355 | CKS1B |  |  | 909 | -0.912 | -0.1815 | Yes |
| 356 | HJURP |  |  | 910 | -0.921 | -0.1770 | Yes |
| 357 | CKS2 |  |  | 911 | -0.922 | -0.1724 | Yes |
| 358 | GTSE1 |  |  | 912 | -0.941 | -0.1677 | Yes |
| 359 | KIF11 |  |  | 913 | -0.957 | -0.1630 | Yes |
| 360 | MYBL2 |  |  | 914 | -0.959 | -0.1582 | Yes |
| 361 | CLSPN |  |  | 916 | -0.964 | -0.1552 | Yes |
| 362 | TYMS |  |  | 917 | -0.965 | -0.1504 | Yes |
| 363 | CENPU |  |  | 918 | -0.971 | -0.1456 | Yes |
| 364 | PRC1 |  |  | 919 | -0.980 | -0.1408 | Yes |
| 365 | MAD2L1 |  |  | 921 | -0.990 | -0.1376 | Yes |
| 366 | UBE2T |  |  | 922 | -0.994 | -0.1327 | Yes |
| 367 | DLGAP5 |  |  | 923 | -0.998 | -0.1278 | Yes |
| 368 | SPC25 |  |  | 924 | -1.033 | -0.1226 | Yes |
| 369 | HIST1H1E |  |  | 925 | -1.049 | -0.1174 | Yes |
| 370 | ESCO2 |  |  | 926 | -1.067 | -0.1121 | Yes |
| 371 | UBE2C |  |  | 928 | -1.108 | -0.1084 | Yes |
| 372 | NUSAP1 |  |  | 929 | -1.112 | -0.1029 | Yes |
| 373 | TPX2 |  |  | 930 | -1.124 | -0.0973 | Yes |
| 374 | NCAPG |  |  | 931 | -1.142 | -0.0917 | Yes |
| 375 | ASPM |  |  | 932 | -1.146 | -0.0860 | Yes |
| 376 | ATAD2 |  |  | 933 | -1.162 | -0.0802 | Yes |
| 377 | CENPF |  |  | 935 | -1.200 | -0.0761 | Yes |
| 378 | HIST1H1C |  |  | 936 | -1.220 | -0.0700 | Yes |
| 379 | HMGB2 |  |  | 937 | -1.238 | -0.0639 | Yes |
| 380 | CDK1 |  |  | 938 | -1.248 | -0.0577 | Yes |
| 381 | FABP5 |  |  | 939 | -1.294 | -0.0513 | Yes |
| 382 | RRM2 |  |  | 940 | -1.403 | -0.0443 | Yes |
| 383 | MKI67 |  |  | 941 | -1.407 | -0.0373 | Yes |
| 384 | TOP2A |  |  | 942 | -1.417 | -0.0303 | Yes |
| 385 | HIST1H1D |  |  | 944 | -1.810 | -0.0231 | Yes |
| 386 | HIST1H1B |  |  | 945 | -1.903 | -0.0137 | Yes |
| 387 | HIST1H4C |  |  | 946 | -2.752 | -0.0000 | Yes |
Table: GSEA details [plain text format]

  

Fig 2: RICHARDS\_NOWAKOWSKI\_SCIENCE\_2017\_IPC\_DIV2\_UPREG: Random ES distribution      
 Gene set null distribution of ES for **RICHARDS\_NOWAKOWSKI\_SCIENCE\_2017\_IPC\_DIV2\_UPREG**
